# Dr. Victor Albert Ellsworth

**Published:** 1915-11

**Authors:** 


**Dr. Victor Albert Ellsworth,** Buffalo 1896, died July 7, aged 69. He was for 21 years superintendent of the Washington Home of Boston and was prominent in temperance work.

